# Biological reasoning…and statistics…

**DOI:** 10.4103/0971-5851.64251

**Published:** 2009

**Authors:** Sudeep Gupta

**Affiliations:** *Editor-in-Chief, Indian Journal of Medical and Paediatric Oncology, Tata Memorial Hospital, Parel, Mumbai, India E-mail: editor@ijmpo.org*

I often hear colleagues, students and even experts express distress at having to reckon with complicated statistical tests when reading published clinical studies. The agony is compounded at having to integrate statistical planning in their own studies, from the calculation of power and sample sizes to correcting for multiple comparisons. How often have I heard the lament – “did the discovery of penicillin require Fleming to deal with ‘t’ tests and hazard functions?” I have often felt the power of this somewhat unthinkingly made statement. This brings me to the fundamental nature of biological reasoning and the place that statistics occupies therein. Indeed, I could generalize these thoughts to all human reasoning.

The origin of human thought and conceptualization is a complex and debatable issue. However, at a simpler level, “reasoning” is derived from “reason,” and implies exploration of the causality of events. Biological reasoning also involves searching (and researching) the causes of biological phenomena, including human diseases. I believe that the origins of these thoughts are essentially non-statistical. For example, the questions “What causes consumption?” or “How can pox be prevented?“ are the result of curiosity into the causes and effects of those syndromes. There is nothing uncertain (statistical) about these *questions*. Similarly, there is nothing statistical (probabilistic) about the question of whether inhibition of angiogenesis will result in regression of tumor growth.

What then is the origin of statistical reasoning in biological thought and experiment? I have implied above that statistical reasoning has something to do with uncertainty. But uncertainty in what? There is surely nothing uncertain about the biological *questions* themselves as I have reasoned above (although, from a philosophical perspective, even this is debatable). The origin of uncertainty is of utmost importance because statistical reasoning is essentially a conceptualization and quantification of this uncertainty. Let me give an example. Suppose there is a large box that contains red and white snooker balls that, apart from their color, are identical in size, shape and feel. Suppose also that there are 50 red and an equal number of white balls. I now request one of my blindfolded students to draw 10 balls sequentially from the box without replacement. It turns out that in this sample, there are seven red and three white balls. What conclusions can be drawn from this experiment? An obvious non-statistical conclusion would be that the box contains 70% red balls and 30% white balls. Although to many of us this seems a rather naïve way of reasoning, it is the way the majority of humans reason in everyday life. When on a particular day I see more sedans than hatchbacks on a particular stretch of my everyday commute to the hospital, I remark to my driving companion that big cars have outnumbered smaller ones in Mumbai. Psephologists routinely pontificate with utmost confidence on primetime television about the relative fortunes of political parties based on the responses of 234 individuals exiting the voting booths. Is there any uncertainty about the *question* of numbers of big versus small cars in Mumbai? Is there any uncertainty about the *question* of which political party will win how many seats in the final tally? There is evidently nothing uncertain about these questions.

The uncertainty in all the above examples is essentially in extrapolation from the sample to the whole population. The uncertainty arises because different samples can be drawn in potentially infinite numbers from commonly sized populations. This is the *raison d’etre* for statistical reasoning in almost all aspects of human thought, including biological questions. There are other motivations for statistical reasoning but they are less important compared to the above. The origin of uncertainty brings me to the formulation of results in biological experiments. Because there will always be some uncertainty in the extrapolation, the result is always a probability. In the first example, there is an “x” probability with “y” confidence that the box has 70% red balls and 30% white ones based on the sample composition. In statistical way of thought, one can never claim with absolute certainty that one number is larger (or smaller) than the other. It turns out, if it is any comfort to biologists, that probabilistic formulation is built into the fabric of nature, exemplified by the fundamental character of the quantum field theory.

This again brings me to the discomfort that otherwise highly intelligent individuals express with statistical reasoning. I believe that human affect is deeply antithetical to uncertainty and probabilities…our species is more comfortable with certainties, including discomfiting ones like death. And average individuals, like most of us, are not alone in our distress…Albert Einstein famously lamented that God cannot play dice. As it turns out…God does play dice.

